# A closer look into the α-helix basin

**DOI:** 10.1038/srep38341

**Published:** 2016-12-05

**Authors:** Boris Haimov, Simcha Srebnik

**Affiliations:** 1 Russell Berrie Nanotechnology Institute, Technion - Israel Institute of Technology, Haifa, 32000, Israel; 2 Department of Chemical Engineering, Technion - Israel Institute of Technology, Haifa, 32000, Israel

## Abstract

α-Helices are the most abundant structures found within proteins and play an important role in the determination of the global structure of proteins and their function. Representation of α-helical structures with the common (*φ, ψ*) dihedrals, as in Ramachandran maps, does not provide informative details regarding the helical structure apart for the abstract geometric meaning of the dihedrals. We present an alternative coordinate system that describes helical conformations in terms of residues per turn (*ρ*) and angle (*ϑ*) between backbone carbonyls relative to the helix direction through an approximate linear transformation between the two coordinates system (*φ, ψ* and *ρ, ϑ*). In this way, valuable information on the helical structure becomes directly available. Analysis of α-helical conformations acquired from the Protein Data Bank (PDB) demonstrates that a conformational energy function of the α-helix backbone can be harmonically approximated on the (*ρ, ϑ*) space, which is not applicable to the (*φ, ψ*) space due to the diagonal distribution of the conformations. The observed trends of helical conformations obtained from the PDB are captured by four conceptual simulations that theoretically examine the effects of residue bulkiness, external electric field, and externally applied mechanical forces. Flory’s isolated pair hypothesis is shown to be partially correct for α-helical conformations.

The middle of the 20^th^ century is considered to be the genesis of structural biology. During this period, Pauling and coworkers discovered the two most fundamental structures found within proteins: the α-helix and the β-sheet^1^,^2^,^3^. This has led to a leap in our understanding of protein structure^4^ and function^5^, and later in folding prediction^6^ and *de novo* design^7^. According to Pauling and coworkers, α-helices have a well-defined structure with constant displacement distances between nitrogen (N) and alpha carbon (Cα) of N-Cα = 1.47 Å, between Cα and carbonyl carbon (C) of Cα-C = 1.53 Å, and C-N = 1.32 Å. The bend angles are fixed at Cα-C-N = 117°, C-N-Cα = 120°, N-Cα-C = 110°, and planar Cα-C-N-Cα = 180° dihedral angle. There are 3.7 amino acid (AA) residues per turn, with a translation of 1.47 Å per residue along the α-helical axis, and a hydrogen bond (HB) between the carbonyl group of every i^th^ residue to the amide group of i + 4^th^ residue with an optimal distance of 2.72 Å between the H-bonded oxygen and nitrogen backbone atoms.

Nearly a decade after the discovery of the α-helix, a systematic tool was developed by Ramachandran and coworkers^8^ for the analysis of the backbone conformation of polypeptides, namely the Ramachandran map. The Ramachandran map allows for distinguishing between regions of similar backbone conformations of polypeptide chains^9^. The Ramachandran map is a plot of dihedral angles *φ* and *ψ*, where *φ* is the C-N-Cα-C dihedral angle and *ψ* is the N-Cα-C-N dihedral angle. An important variation of the Ramachandran analysis is the neighbor dependent analysis. Neighbor dependent studies allow to take into account the effects of the neighbors on the (*φ, ψ*) propensity of a given AA^10^,^11^,^12^. One of the interesting conclusions drawn from Ramachandran’s pioneering study was that more than half of the regions on the Ramachandran map are sterically inaccessible. The understanding of the inaccessible and accessible Ramachandran regions is crucial for the understanding of the distribution of the empirically determined backbone conformations.

The Ramachandran map opened the doors for a wide variety of empirical and theoretical conformational studies of α-helical polypeptides. Empirical determination of α-helix structures demonstrated a diagonal distribution of the (*φ, ψ*) pairs^13^,^14^. Since the α-helix basin is found near a sterically inaccessible region near the origin and is distributed along a diagonal, it was believed that the cause for the observed distributions is its location on the Ramachandran map^13^. Earlier studies carried out by Scheraga and coworkers^15^,^16^,^17^,^18^, also pointed out the relevance of the diagonals when dealing with α-helical conformations. A later study revealed that geometrical constraints of α-helical HBs are likely to be the reason for the observed diagonal distributions^14^. Further empirical studies^19^,^20^ showed that the different AAs are found in different proportions within α-helices due to the energetic cost for inclusion of some given AA within the α-helix. The latter allowed the definition of α-helix propensities for the different AAs. Accordingly, MET, ALA, LEU, GLU (E), and LYS (K) (or shortly MALEK in one-letter AA codes) are the AAs with the highest α-helix propensity while PRO and GLY are with the lowest.

Following these advances, many efforts have been invested in the study of α-helices, however, little was done to understand the conformations of α-helices within the α-helix basin. Thus, the purpose of this study is to provide a deeper look into the different conformations of α-helices.

## Results

**Figure 1:**
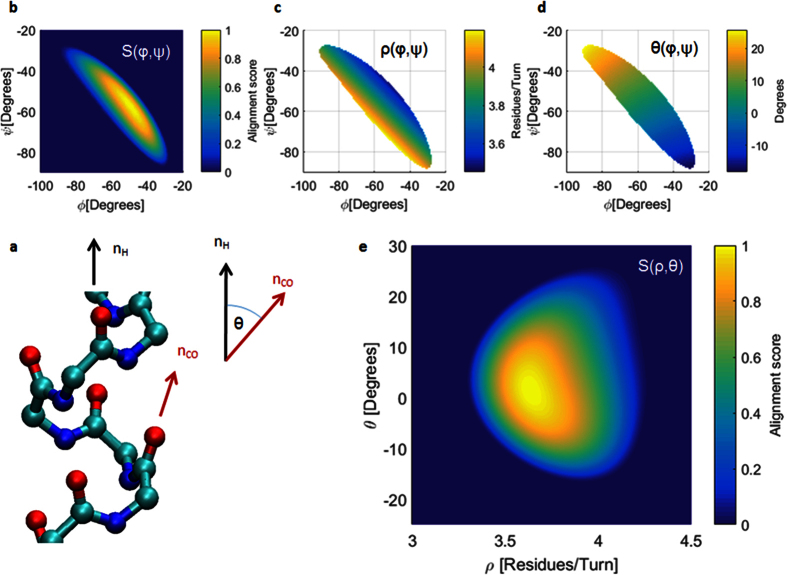
Derivation of the alternative (*ρ, ϑ*) coordinates system for the α-helix basin. (**a**) Definition of the *ϑ* angle: n_H_ is the helix direction normal and n_CO_ is the carbonyl oxygen normal, *ϑ* is positive when n_CO_ points outward the helix center and negative otherwise. Oxygen atoms are red, nitrogen atoms are blue, and carbon atoms are cyan. (**b**) Analytical determination of the α-helix basin by hydrogen bond (HB) alignment score *S*: no HB alignment for regions with *S* = *0* and maximal alignment for regions with *S* = *1*. The optimum of the HB alignment is found on the diagonal *φ* + *ψ* = −*107.8*° at (*φ*_*M*_, *ψ*_*M*_) = (−*49.7*°, −*58.1*°). (**c**) Calculated number of residues per single α-helix turn for regions with *S* > *0*. (**d**) Calculated angle *ϑ* between a CO normal and the helix normal for regions with *S* > *0*. The approximate linear dependence of (*ρ, ϑ*) on (*φ, ψ*) may be clearly seen in both (**c** and **d**). (**e**) The α-helix basin HB alignment score on the linearly approximated (*ρ, ϑ*) coordinates system. The optimal conformation for HB alignment on the (*ρ, ϑ*) space is found approximately at *ρ* = *3.62* ± *0.2* [*Res/Turn*] and *ϑ* = *1.1* ± *2.4*°. PDB animations that demonstrate the change of *ρ* and *ϑ* in α-helices can be found in supplementary material SM1_rho.pdb and SM2_theta.pdb, respectively.

We wish to find a mathematical relation between (*φ, ψ*) and (*ρ, ϑ*) coordinate systems, where (*φ, ψ*) are the commonly used dihedral angles, *ρ* is the number of residues per single turn of the α-helix, and *ϑ* is the angle between a backbone carbonyl (CO) normal of the given residue relative to the normal of the α-helix direction (*ϑ* is positive for CO normal pointing outwards from the α-helix center) as illustrated in Fig. 1a. The relation between *ρ* and *ϑ* as a function of (*φ, ψ*) is shown in Fig. 1c and d, respectively. The analytical determination of the α-helix basin was done by using the HB alignment score *S*, such that regions with *S* > *0* are treated as the α-helix basin in this study. Figure 1b illustrates the evaluated score *S* as a function of (*φ, ψ*), and presents visually the shape and the location of the α-helix basin. Next, we derived a linear transformation from (*φ, ψ*) coordinates to (*ρ, ϑ*) coordinates with the following set of equations:


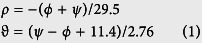


where the errors of *ρ* and *ϑ* are Δ*ρ* < *0.2* [*Res/Turn*] and Δ*ϑ* < *2.4*° for *S* > *0*. Figure 1e presents the alignment score *S* on the resulting (*ρ, ϑ*) space.

**Figure 2:**
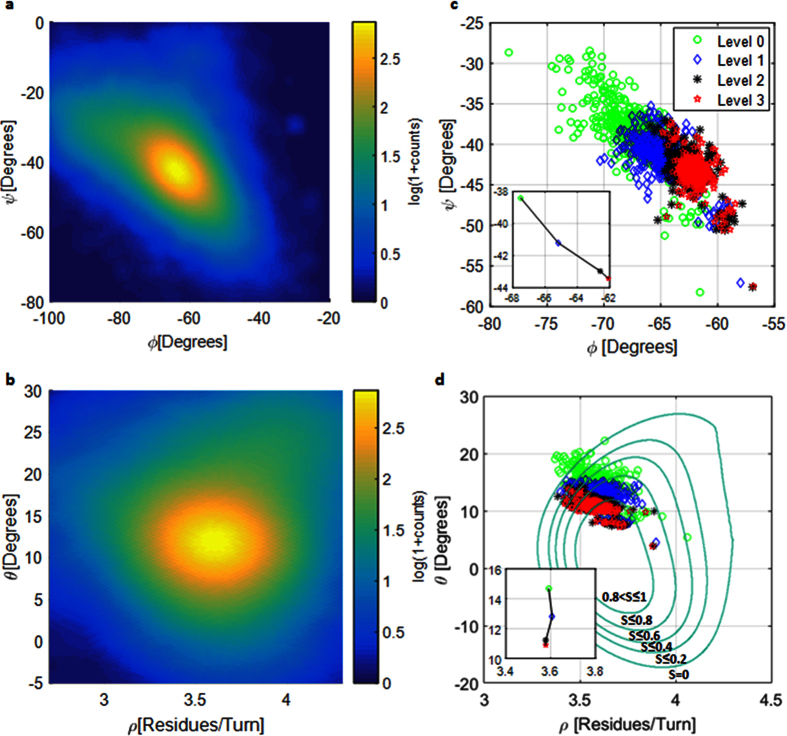
(**a**) Typical diagonal distribution of Alanine->Alanine transitions in alpha helices on (*φ, ψ*) coordinates system. (**b**) Typical distribution of Alanine->Alanine on the (*ρ, ϑ*) coordinates system. (**c**) Mean values of the 400 possible amino acid transitions for the four filtering levels on (*φ, ψ*) coordinates system. The inset shows the linear migration towards high *φ* values and low *ψ* values with the increasing filtering level (points in the inset stand for the overall mean value at the given filtering level). (**d**) The mean values of the 400 transitional pairs from B on the (*ρ, ϑ*) coordinates system at four filtering levels. Green contours present the hydrogen bond alignment score boundaries for *S* = *0, 0.2, 0.4, 0.6*, and *0.8*. The inset shows the migration towards low *ϑ* values and almost no change of ρ with the increasing filtering level (points in the inset stand for the overall mean value at the given filtering level). The samples for (**c** and **d**) are marked according to the filtering level: green circles for Level 0, blue diamonds for Level 1, black asterisks for Level 2, and red stars for Level 3.

In this study we distinguish between 400 naturally occurring AA pairs which we name as transitions. By naming pairs as transitions we actually emphasize the importance of directionality when dealing with polypeptides. On the (*φ, ψ*) space a transitional conformation is the (*φ, ψ*) pair describing the conformation of the transition from AA_X_ to AA_Y_. For a transition advancing from N to C terminus with the following backbone atoms: N_X_-Cα_X_-C_X_-N_Y_-Cα_Y_-C_Y_, we define the transitional *ψ* = *ψ*_X−>Y_ dihedral as N_X_-Cα_X_-C_X_-N_Y_ and the transitional *φ* = *φ*_X−>Y_ dihedral as C_X_-N_Y_-Cα_Y_-C_Y_. This naming convention is used to describe the conformation of the α-helix backbone. Figure 2a presents the distribution of all the sampled ALA−>ALA transitions on the (*φ, ψ*) coordinates system. It is clearly observed that the (*φ, ψ*) distribution is along the *φ* + *ψ* = *const* diagonal, which is a result of the HBs along the α-helix backbone^14^,^21^ and the sterically inaccessible regions near the α-helix basin^13^,^14^. Figure 2b presents the typical distribution of all the sampled ALA−>ALA transitions on the (*ρ, ϑ*) coordinates system. By observing Fig. 2b we immediately conclude that ALA−>ALA transitions found in PDB helices contain an average of *3.6* [*Res/Turn*] and a *ϑ* angle of about *12*° relative to the helix direction normal in agreement to previous reports^22^,^23^,^24^,^25^. The latter is a validation of the (*φ, ψ* to *ρ, ϑ*) transformation applied on the PDB data, presented in Equation 1. All other transitions demonstrate very similar distributions to those of ALA−>ALA with the exception of PRO and GLY that will be discussed later in this study. The difference between the different transitions is in the mean value of the specific distribution. An interesting result presented in Fig. 2b is the symmetrical Gaussian-like fluctuations on the alternative coordinates system. The fluctuations of the measured values are the result of measurement, thermal, and other sources of noise. The symmetrical fluctuation pattern allows drawing the following conclusion: by focusing on some circular contour on Fig. 2b, the green one for instance, we deduce that the energy to cause a shift of ~*0.8* [*Res/Turn*] is the same as the energy to cause a shift of ~*15*° of the *ϑ* angle. The implication of this observation will be further discussed within the Heterogeneous transitions section.

**Figure 3:**
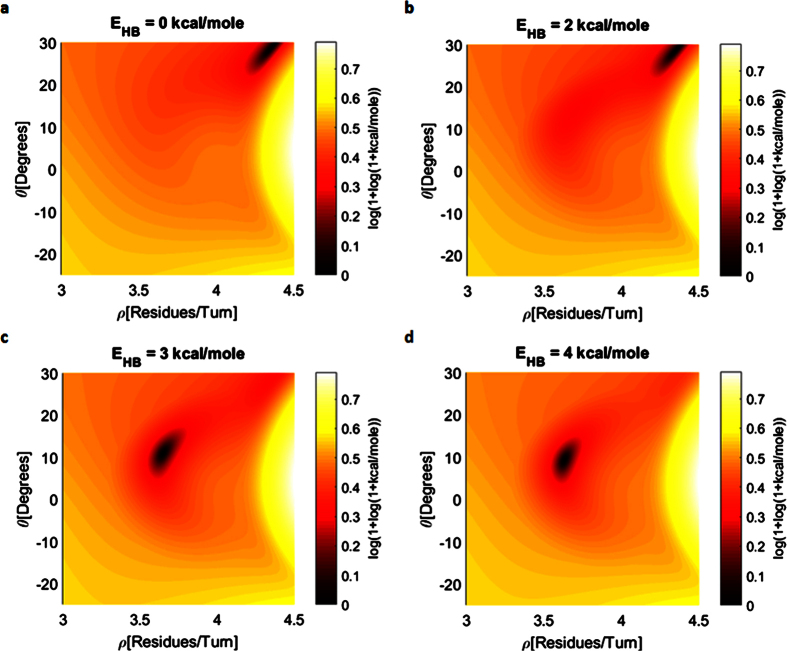
The role of hydrogen bond interactions (HBI) in the determination of the optimal conformation of the α-helix backbone. (**a**) Without HBI the optimal conformation is found at *ρ* = *4.35* [*Res/Turn*] and *ϑ* = *28.7*°. The optimal conformation in this case is determined by van der Waals steric interactions. (**b**) Inclusion of weak HBI with magnitudes of up to *E*_*HB*_ = *2* [*kCal/mole*] do not demonstrate a significant shift of the optimal conformation and remain at *ρ* = *4.35* [*Res/Turn*] and *ϑ* = *28.7*°. (**c**) Inclusion of HBI with a magnitude of *E*_*HB*_ = *3* [*kCal/mole*] shift the optimal conformation to *ρ* = *3.65* [*Res/Turn*] and *ϑ* = *10.9*°. (**d**) Inclusion of HBI with a magnitude of *E*_*HB*_ = *4* [*kCal/mole*] shift the optimal conformation to *ρ* = *3.63* [*Res/Turn*] and *ϑ* = *9.3*°. According to the literature, common HBI magnitudes for the α-helix backbone are from *2* [*kCal/mole*] to *6* [*kCal/mole*]. The intermediate value of *E*_*HB*_ = *4* [*kCal/mole*] predicts that the optimal conformation is around *ρ* = *3.63* [*Res/Turn*] and *ϑ* = *9.3*° for α-helix backbones. Because of the high difference between the maximal and the minimal energy values, a logarithmic function of the form log(*1* + (log(*1* + *E*))) was applied on all the resulting energy values to allow better visualization of the energy maps.

The mean value of each of the 400 naturally occurring transitions is displayed in Fig. 2c for every one of the 4 filtering levels. The inset of Fig. 2c shows the mean value of all of these transitions for the given filtering level. We clearly observe the migration of the transitional (*φ, ψ*) pairs towards higher *φ* values and lower *ψ* values along the *φ* + *ψ* = *const* diagonal, which suggests that for helices with better aligned HBs we should expect higher *φ* values and lower *ψ* values. This raises the question of how much we can increase *φ* and decrease *ψ* along the *φ* + *ψ* = *const* diagonal such that the polypeptide will remain in its α-helical shape. The answer is found within Equation 3 (in Methods) that shows that the optimum of the HB alignment equation is found on the line *φ* + *ψ* = −*107.8*° at (*φ*_*M*_, *ψ*_*M*_) = (−*49.7*°, −*58.1*°). Figure 2d presents the same transitions as in Fig. 2c but on the (*ρ, ϑ*) coordinates system. A clear migration towards regions of high alignment score is observed with increasing filtering level. Furthermore, a very weak change is observed from filtering level 2 and level 3 which suggests that the maximally aligned HB conformation at (*ρ, ϑ*) ~ (*3.62* [*Res/Turn*], *0*°) is hard to reach. In Fig. 3 we show that the conformational optimum of (*ρ, ϑ*) = (*3.63* [*Res/Turn*], *9.3*°) is a direct result of HB interactions on the order of *4* [*kCal/mole*]. Having the distributions of the 400 naturally occurring transitions, we divide the analysis into two parts: 20 homogeneous (same AAs) and 380 heterogeneous (different AAs) transitions analysis.

### Homogeneous transitions

**Figure 4:**
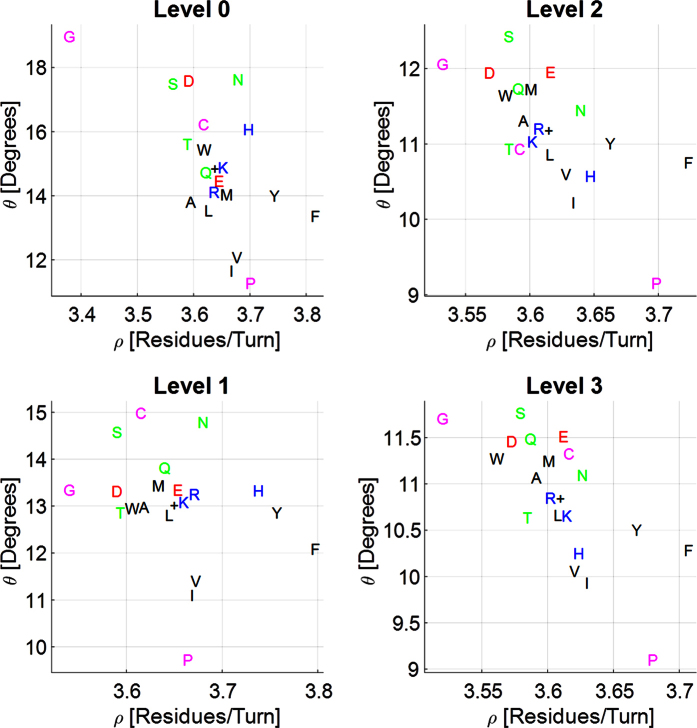
Homogeneous transitions for the four filtering levels. Single letter amino acid (AA) codes are placed on the mean calculated conformation. Letters are colored according to the AA group: green for uncharged polar, red for acidic, blue for basic, black for hydrophobic, magenta for special. The black + symbols are placed on the mean overall conformation for every filtering level. The four filtering levels are of increasing levels of order, where Level 0 filtering is the less ordered and might even include non-helical conformations. Level 1 filtering includes only α-helical regions but with possible kinks and other types of deformations. Level 2 filtering includes a subset of conformations that must satisfy hydrogen bond (HB) alignment criterion with a weak threshold. Level 3 filtering is the most ordered filtering criterion with a strong HB alignment threshold. MALEK are the AAs with the highest helix propensity and are closely spread around the center of each filtering level, especially in filtering Level 0 and Level 1. G and P are known for their low helix propensity and show a clear tendency to stay away from the overall mean helical behavior.

Figure 4 presents the mean (*ρ, ϑ*) values of the 20 possible homogeneous transitions at the four filtering levels, the higher the level the better aligned the backbone of the helix. MALEK are known as the AAs with the highest helical propensities^20^, and as indeed may be observed in Fig. 4, MALEK are closely spread around the center of each filtering level, especially in filtering Level 0 and Level 1. This suggests that MALEK are helically well aligned AAs and practically dictate the location of the mean helix conformation. It is clearly observed that GLY transitions are always the leftmost with the lowest amount of residues per turn and PRO transitions are always at the bottom with lowest *ϑ*. GLY and PRO have a clear tendency to stay away from the overall mean helical behavior (which is denoted by the + symbol on every plot), possibly due to the high energetic cost to include GLY and PRO within α-helices^19^,^20^. However, GLY and PRO differ in their local effect on helix geometry. GLY strongly decreases the number of residues per turn and keeps *ϑ* above the overall average as is observed in all filtering levels. PRO, on the other hand, keeps the number of residues per turn above the overall mean and strongly decreases *ϑ*, as observed in all the filtering levels.

Interestingly, the basic AAs ARG (R) and LYS (K) demonstrate a conformational behavior that is very close to the overall mean conformation in all the filtering levels. The basic AA HIS, demonstrates the highest amount of residues per turn among all the basic AAs and the propensity to stay above the overall mean amount of residues per turn in all the filtering levels. Similarly, the polar uncharged AAs SER, THR, and GLN (Q) are always with less residues per turn than the overall mean, while the amount of residues per turn for ASN (N) is always above the overall mean.

**Figure 5:**
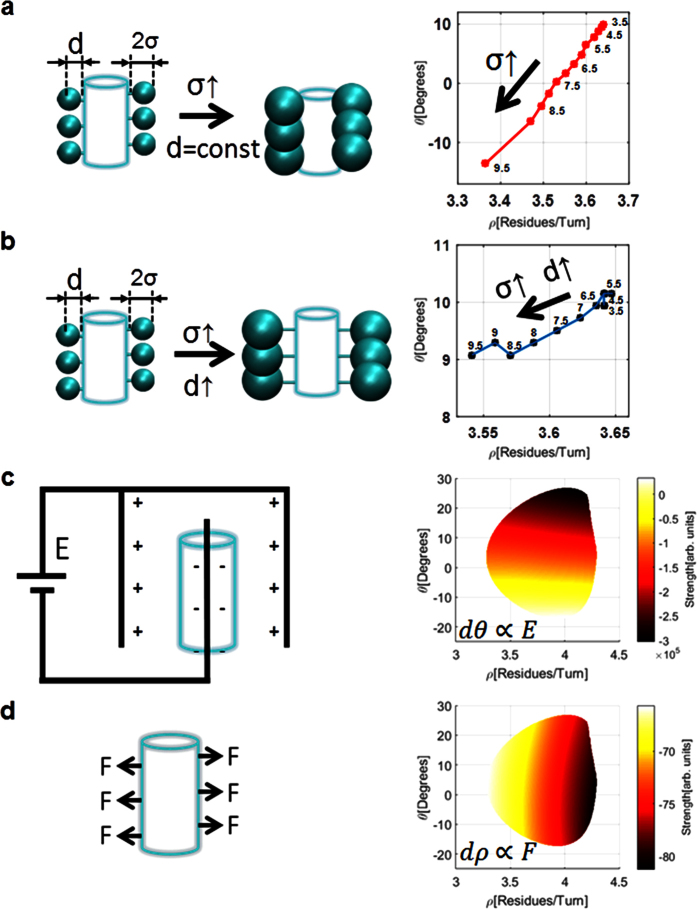
Illustration of the four conceptual simulations and the resulting effects on the helical conformation. (**a**) Conceptual Simulation 1: Residues with increasing bulkiness near the backbone result in a strong decrease of both *ϑ* and *ρ*. (**b**) Conceptual Simulation 2: Residues with increasing bulkiness far from the backbone result in a decrease of ρ and a negligible decrease in *ϑ*. (**c**) Conceptual Simulation 3: Externally applied electric field result in an increase of *ϑ* proportional to the electric field magnitude. (**d**) Conceptual Simulation 4: Externally applied stretching force result in an increase of ρ proportional to the magnitude of the applied force. Cyan cylinders represent α-helix backbones. Value of vdW radius (σ) in Ångströms is shown on plots **a** and **b** and was increased from *3.5* *Å* to *9.5* *Å* in steps of *0.5* *Å*. The bond length *d* was set to *1.54* *Å* in Conceptual Simulation 1, and was maintained at *σ* *−* *1.96* *Å* in Conceptual Simulation 2.

Both ILE and VAL demonstrate a strong propensity to lower *ϑ*. These AAs are the only residues with two carbons at the *γ* position, which raises the question whether they are the reason for the observed lower *ϑ* angle. To assert this premise we performed Conceptual Simulations 1 and 2 as described in the Methods section and depicted in Fig. 5. The results of the inflated virtual residue in Fig. 5a shows that bulkiness near the helix backbone strongly decrease *ϑ* and also decrease *ρ*. Thus, we may deduce that since VAL is less bulky than ILE it decreases *ϑ* less as predicted by the conceptual simulations.

THR is a polar and uncharged AA that differs from VAL by the oxygen atom on the *γ* position, otherwise VAL and THR are sterically very similar and might be expected to behave similarly. As may be observed in Fig. 4, THR demonstrates considerably lower *ϑ* angle than the other uncharged AAs (SER, ASN (N), and GLN (Q)) in all the filtering levels, with the exception of GLN (Q) in Level 0. The latter may be because Level 0 may contain non-α-helical conformations, and because of the polar nature of the THR and GLN (Q) residues. Previous reports suggest that the bulky residues stabilize the α-helix HBs and shield them from surrounding water molecules^23^,^25^. Our observations of bulkiness in the vicinity of the backbone for ILE, VAL, and in some cases for THR confirm that the shielding of the α-helix backbone occurs via the decrease of *ϑ*, since increased values of *ϑ* suggest the existence of nearby water molecules that destabilize the α-helical HBs^22^. The exceptions of THR in the lower filters might be explained by its polar nature.

The results for Conceptual Simulation 2 that focus on the effect of increasing distance of the virtual residue, presented in Fig. 5b, suggest that almost no conformational changes take place when the residue is inflated up to the critical value *σ*_*C*_ = *6* *Å*. Above σ_C_ we observe a decrease in the number of residues per turn while *ϑ* remains nearly constant. By focusing on the most bulky AAs PHE (F), TYR (Y), and TRP (W) (in increasing order of bulkiness, respectively) in Fig. 4 we indeed observe a decrease in the amount of residues per turn with the increase of bulkiness, as is confirmed by the conceptual simulation. In addition, two mismatches are evident: (1) FYW are expected to be to the left of MALEK but observed with an increased *ρ* (shifted rightwards in Fig. 4), and (2) FYW are expected to demonstrate a slight decrease of *ϑ* with the increase of bulkiness but demonstrates an increase of *ϑ* with the increase of bulkiness. The reason for the observed mismatches might be the naïve nature of the performed conceptual simulation that does not take into account interactions other than steric.

Since α-helices are not isolated structures and probably interact with the surrounding environment, we performed two additional simulations to understand the dependence of the helical structure on the surrounding forces. On the atomic scale, charged particles may induce local effects similar to those applied by an electric field^26^,^27^,^28^. Thus, we tested the effect of an applied electric field as illustrated in Fig. 5c. As clearly observed from the resulting plot, the electric field promotes a change of *ϑ*, and has a negligible effect on the amount of residues per turn, or literally the change in *ϑ* is proportional to the magnitude of the applied electric field. The small but still present change in the *ρ* axis is due to the unequal magnitudes of the partial charges of the carbonyl oxygens (−*0.5*) and amide hydrogens (+*0.33*). For the hypothetical case where both of the partial charges were with the same absolute magnitude we would expect no dependency of the electric potential on the *ρ* axis.

In the last simulation shown in Fig. 5d we measure the contribution of an external stretching force applied only on Cα’s of the helix backbone. The plot shows the contribution of the external stretching force to the total conformational energy of the α-helix backbone. As clearly observed, stretching forces encourage the change of the number of residues per turn with a negligible change of *ϑ*, or literally the change in *ρ* is proportional to the magnitude of the externally applied force. The observed behavior demonstrates practically no change in *ϑ* since change in *ϑ* results in energetically unfavorable misalignment of the HBs. In both conceptual simulations 3 and 4, changing the sign of the electric field (direction of the applied force) will result in exactly the opposite change of *ϑ (ρ*).

### Heterogeneous transitions

A heterogeneous transition is a transition AA_1_−>AA_2_ where AA_1_ ≠ AA_2_ with a total of possible 380 heterogeneous transitions for the 20 common AAs. Flory’s isolated pair hypothesis (IPH)^29^ which was shown to be generally incorrect^30^,^31^,^32^,^33^ states that the (*φ, ψ*) pair is independent of its neighbors. Interestingly, IPH was never discussed in detail specifically for the α-helix basin, probably because of the difficulties distinguishing between one helix and another. Our approach of analyzing conformations on the (*ϑ, ρ*) space allows studying the differences even between very similar α-helical conformations and will be used test the validity of IPH for the α-helix basin. Before approaching IPH we will focus on another important question which is directly related to IPH: Can we predict the conformational behavior of a heterogeneous transition AA_1_−>AA_2_ from the arithmetic mean of the homogeneous transitions: AA_1_−>AA_1_ and AA_2_−>AA_2_? To perform the comparison between a measured conformation to some predicted conformation we calculate the energetic cost of the predicted conformation relative to the measured conformation. We deduced earlier that the energy required to shift *~0.8* [*Res/Turn*] is the same energy required to shift ~*15*° of the *ϑ* angle for the green contour visually presented in Fig. 2b. To find more precise values of the shift, we repeated the latter calculation and found that 50% of all the measured transitions are confined *within* (Δ*ϑ*, Δ*ρ*) ≈ (*13.5, 0.8*), (*10.5, 0.6*), (*9.6, 0.6*) and (*8.7, 0.5*), for the four filtering levels 0–3, respectively, with an average of (〈Δ*ϑ*〉, 〈Δ*ρ*〉) ≈ (*10.6*°, *0.63* [*Res/Turn*]). The rationale behind such averaging is to give more weight to the more aligned helical structures (higher filtering levels). If we define a single Energy Unit (*EU*) as the energy required to a shift of Δ*ϑ* = *1*°, and a conversion ratio *K* = 〈Δ*ϑ*〉/〈Δ*ρ*〉 ≈ *16.8* [°*Turn/Res*], we may use the following harmonic energy expression to approximate the energy of some given conformation:


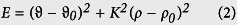


where *ϑ*_*0*_ and *ρ*_*0*_ represent the minimum energy conformation for some given transition. Since both 〈Δ*ϑ*〉 and 〈Δ*ρ*〉 are the average confinement values for half of the measured transitions we deduce that the border energy of the confined transitions is *E*_*0.5*_ = (〈Δ*ϑ*〉/*2*)^*2*^ = *K*^*2*^(〈Δ*ρ*〉/*2*)^*2*^ ≈ *28* [*EU*], which is approximately the energy of the green contour in Fig. 2b.

Table 1 presents the energy difference between the measured conformations to the predicted conformations for the heterogeneous transitions for filtering Level 1 (tables for other levels may be found in Supporting Information). The conformations were predicted by calculating the arithmetic mean of the homogeneous conformations. The energy differences were calculated using Equation 2, by defining the measured conformation as the minimum energy conformation. The results suggest that the mean energy difference of all cases is ~*3* [*EU*], that the highest energy differences are observed for transitions from PRO (AA_PRO_−>AA_X_) and to PRO (AA_X−>_AA_PRO_), and that in most cases the transitions carry an asymmetric nature, i.e. Δ*E*_*AA1*−>*AA2*_ *≠* Δ*E*_*AA2*−>*AA1*_. The asymmetry issue is enough to conclude that in most cases predicted conformations will differ from the real conformations. Nevertheless, if energy differences are tolerable, the prediction of heterogeneous conformations may sometimes be useful especially when excluding PRO. In case the tolerance is set to a very small value of *1* [*EU*] we find that 45% (171 transitions out of 380) of the heterogeneous transitions are predictable by homogeneous averaging, and in case the tolerance is set to the half population boundary energy *E*_*0.5*_ = *28* [*EU*], *97*% (375 out of 380) of the heterogeneous transitions are predictable by homogeneous averaging. If IPH was absolutely correct than we would expect that all the values presented in Table 1 would equal to 0. Table 1 is actually a proof that IPH is incorrect when no tolerance in energy difference is allowed, however when introducing such tolerance, IPH becomes *45%* correct for *1* [*EU*] tolerance and *97*% correct for *28* [*EU*] (which is approximately the energy of the green contour in Fig. 2) tolerance as explained above for Level 1 filtering (the percentages increase with increased filtering). In addition, the asymmetric nature of the transitions justifies the transitional analysis approach that was done in this study and stresses that previous efforts of analyzing α-helical conformations lack important transitional information.

A possible explanation to the observed deviation between heterogeneous transitions to their homogeneous average is the interaction between residues – residues that do not interact chemically, sterically, or in any other way are expected to demonstrate stochastic conformational behavior, i.e. the average conformation of two non-interacting AAs are expected to be equal to the observed heterogeneous conformation. Thus, we can conclude that the higher the energy difference between the heterogeneous conformation to the average homogeneous, the higher the interaction between the residues with the exception of PRO that may result in high deviations because of its limited degrees of freedom.

## Discussion

By representing helical structures on the (*ρ, ϑ*) space we attribute a meaning to the 2-dimensional representation of the α-helix in terms of residues per turn (*ρ*) and the CO angle of backbone carbonyls relative to the helix direction vector (*ϑ*). It was shown that a simple linear transformation allows for switching between (*φ, ψ*) and (*ρ, ϑ*) spaces, giving freedom of choice for the desired representation space. The transformation was validated by comparing our observations with those found in the literature. By using our new (*ρ, ϑ*) space we were able to deduce that: (1) *50%* of α-helix conformations found in PDB are confined in average within (〈Δ*ϑ*〉, 〈Δ*ρ*〉) *≈* (*10.6*°, *0.63* [*Res/Turn*]). (2) The energy required to shift the conformation by Δϑ = 16.8° is the same energy required to shift the conformation by Δ*ρ* = *1* [*Res/Turn*] within the α-helix basin. (3) Residues with bulkiness near to the helix backbone (the case of VAL, ILE, and THR) decrease *ϑ* stronger than other residues. (4) Residues with bulkiness far from the helix backbone (the case of PHE (F), TYR (Y), and THR (W)) demonstrate a decrease in *ρ* with increased bulkiness. (5) An environment with charged particles affects primarily *ϑ*. (6) External stretching/squeezing forces affect *ρ*. Furthermore, It was shown that representation of helical structure on the (*ρ, ϑ*) space has the advantage of easily calculating the conformational energy of any given α-helix, which is not applicable on the (*φ, ψ*) space. The latter allowed to approach Flory’s IPH problem and to draw relevant conclusions. This study presented the α-helix basin from a novel perspective and resolutions that were not previously available.

## Methods

This study is performed in three parts: (1) Derivation of the alternative coordinate system (*ρ, ϑ*) and its relation to the commonly used coordinates system (*φ, ψ*), (2) Statistical study of α-helical conformations found in PDB, and (3) Conceptual simulations to understand the conformational tendencies of α-helices found in PDB. In the 1^st^ part we use the base simulation model of the α-helix backbone to calculate the alignment score of HBs. We use the conformational sweep method to analytically determine the alignment score for all the possible α-helical conformations. One of the purposes of HBs alignment score is to analytically determine whether any given conformation is α-helical or not. Finally, we use the HBs alignment score to calculate the conformational backbone energy of any given α-helix. In the 2^nd^ part we explain how we organized the PDB data from less ordered helical structures to more ordered helical structures and how the PDB redundancy issue was treated. In the 3^rd^ part we adjust the base simulation model to conceptually demonstrate how the shape of the residue affects the helical conformation, and how the environment affects the helical shape by applying external electric field, and external mechanical forces. In-house software was developed under C++ and Matlab^TM^ to perform the simulations and the analysis of the PDB data. Visual Molecular Dynamics (VMD)^34^ was used for the 3D visualization of molecular structures.

### Base simulation model of the α-helix backbone

The base model consists of 30 AA-long polypeptide backbone (without residues) and was used as the basis for the calculations carried out in this study. All the geometrical values for the base model were sampled directly from PDB. The resulting values used for the base model are: constant distances N-Cα = 1.46 Å, Cα-C = 1.52 Å, C-N = 1.33 Å, C-O = 1.23 Å, constant bend angles Cα-C-N = 117°, C-N-Cα = 121°, N-Cα-C = 111°, and a constant dihedral Cα-C-N-Cα = 180°. The values confirmed the proper function of the custom software designed for the sampling and analysis used in this study. The positions of the carbonyl oxygens assumed the Cα, C, O, N backbone atoms on the same plane with equal bend angles Cα-C-O = N-C-O. No hydrogen atoms were included in the base model.

### Conformational sweep

Conformational sweep is an important method used in this study to evaluate properties of the α-helical conformation. If *P* is some property of interest that is a function of the α-helical conformation, then by using conformational sweep over a binned space (*φ, ψ* space for instance) we perform the calculation of *P* on every binned point within the space of interest and get a resulting map *P (space of interest*).

### Scoring the α-helical hydrogen bond alignment

HBs play a key role in the formation of the α-helical shape^1^,^21^,^35^,^36^,^37^,^38^,^39^. Thus, the alignment score of HBs is used to analytically determine the quality of some given α-helical polypeptide segment by using the following scoring function:


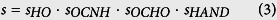


where *s*_*HO*_ = *max(0, 1* − (*dst(HO*) − *1.9*)^*2*^/*1.2*^*2*^) is the harmonic sub-score of the H-O distance with *H* being the hydrogen of the i + 4^th^ residue nitrogen and *O* being the i^th^ residue oxygen. The optimal distance of *1.9* *Å* and a fluctuation range of ±*0.6* *Å* were picked for the harmonic function. *s*_*OCNH*_ = *max(0, OC·NH*) is the angular alignment sub-score of the *OC* normal of the i^th^ residue with the *NH* normal of the i + 4^th^ residue. *s*_*OCHO*_ = *max(0, OC·HO*) is the angular alignment sub-score of the *OC* normal of the i^th^ residue with *HO* normal, where *H* is the hydrogen of the i + 4^th^ nitrogen and *O* is the i^th^ oxygen. *s*_*HAND*_ equals to 1 for right handed α-helices and 0 otherwise. All the sub-scores *s*_*HO*_, *s*_*OCNH*_, *s*_*OCHO*_, and *s*_*HAND*_ range from 0 to 1 thus the total score s range from 0 to 1, with *s* = *0* meaning no HB alignment detected and *s* = *1* meaning the best alignment detected. The total score *0* ≤ *S* ≤ *1* for a given polypeptide chain measures the mean alignment of all possible i^th^ to i + 4^th^ HBs, such that *S* = 〈*s*〉.

### Calculation of (*ρ, ϑ*) given (*φ, ψ*)

Given (*φ, ψ*) we first calculate the Cartesian coordinates of every atom of the base model. To calculate the number of residues per turn for the base model, we evaluate the rotation angle *r*_*i,i*+*1*_ between every two consecutive Cα’s and calculate the cumulative rotation angle as 
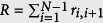
 for *N* residues. Next we calculate the amount of residues per turn as *ρ* = *2πN/R*. To calculate *ϑ*, we first calculate the helix direction vector as 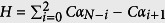, where *Cα*_*i*_ is the position of i^th^ Cα on the Cartesian space. Next, we find the helix normal *n*_*H*_ = *H*/||*H*||. Letting *C*_*i*_ and *O*_*i*_ be the Cartesian positions of the i^th^ residue carbonyl carbon and oxygen atoms, respectively, we find the vector *Q*_*i*_ which is pointing from the nearest point on the helix vector toward *C*_*i*_, and accordingly its normal *q*_*i*_ = *Q*_*i*_/||*Q*_*i*_||. Finally, we calculate 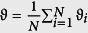, where *ϑ*_*i*_ = *a* *sin* (−*q*_*i*_*·n*_*COi*_), and *n*_*COi*_ = *CO*_*i*_/||*CO*_*i*_|| is the normal of the *CO* vector of i^th^ residue given by *CO*_*i*_ = *C*_*i*_ − *O*_*i*_.

### Conformational energy of the α-helix backbone

The optimal conformation according to the scoring function illustrated in Fig. 1e is evaluated at *ρ* = *3.62* [*Res/Turn*] and *ϑ* = *1.1*°. The non-zero value of *ϑ* is due to the binning of the (*ρ, ϑ*) space and the theoretical expected value of *ϑ* for the optimal conformation should be *ϑ*  = *0*°. To find the optimal α-helix conformation with the inclusion of steric energy we introduced the vdW interactions in the form of Lennard-Jones (LJ) 12–6 potential to the HB energy. Since the value of HB is usually in the range of *2* to *6* [*kCal/mole*]^30^,^38^,^39^,^40^, we used a value of *4* [*kCal/mole*] in our numerical calculations and expressed the HB energy as *E*_*HB*_ = −*4S* [*kCal/mole*]. In addition, we used OPLS force field^41^,^42^ parameters with geometric mixing rules for the calculation of the vdW backbone energy: *σ(N, CA, C, O*) = (*3.25, 3.5, 3.75, 2.96*) [*Å*], and *ε(N, CA, C, O*) = (*0.17, 0.066, 0.105, 0.21*) [*kCal/mole*].

### PDB data sampling at four filtering levels

PDB is a worldwide rapidly growing database of biological structures that is open to public access^43^,^44^. It includes proteins acquired by different techniques with X-RAY and NMR protein structure acquisition techniques used in 99% of the cases. To date, more than 100 K empirically determined structures are available on PDB. Transitional Ramachandran (*φ, ψ*) pairs of the common 400 AA transitions found in PDB were sampled and filtered according to four levels: Level 0 filter checks whether the (*φ, ψ*) pair is found within a predefined window where −*100° *< *φ *< −*20°* and −*80°* < *ψ* < *0°*. Level 1 filter checks whether the given residue pair is found within α-helical regions as determined within the PDB file usually with PROMOTIF based on DSSP^37^. Level 2 filter is the custom filter based on Equation 3 that checks the satisfactory of HBs alignment with a weak threshold of *S* ≥ *0.01*. Level 3 filter is the same custom filter with a stronger threshold of *S* ≥ *0.5*. The four filtering levels are of increasing levels of order, where Level 0 filtering is the less ordered and might even include non-helical conformations. Level 1 filtering includes only α-helical regions but with possible kinks and other types of deformations. Level 2 filtering includes a subset of conformations that must satisfy HB alignment criterion with a weak threshold. Level 3 filtering is the most ordered filtering criterion with a strong HB alignment threshold. The transitional conformation (*φ, ψ*) pairs were sampled into 400 2D histograms.

### PDB dataset resolution and redundancy treatment

In this study we sampled all of the available conformational data found in PDB for α-helices. We did not introduce any resolution limit because of the stochastic nature of the sampled data in PDB, and since we believe that important conformational data might be found even in low resolution measurements^45^. To reduce the effects of PDB redundancy we applied a logarithmic function on the resulting distributions. In addition, the distribution of every transition was normalized by its area, such that the sum of all the possible conformations for a given transition equals to 1.

### Conceptual simulation 1: residues with near bulkiness

The goal of this simulation is to demonstrate how bulky groups that are close to the α-helix backbone affect the conformation of the α-helical structure. We introduced virtual residues to every Cα on the backbone of the base model. The virtual residue is similar to ALA residue, but is attached perpendicular to the α-helix backbone while ignoring the hydrogen at the Cα position as is found in real residues. This was done to simulate the average space covered by the many possible conformations of any given residue and to allow a conceptual analysis of what happens to the α-helix when the residue is inflated. The virtual residue was maintained at a constant distance of *1.54* *Å* from the Cα in one case while the vdW radius (*σ*) of the virtual residue was changed from *3.5* *Å* to *9.5* *Å* with steps of *0.5* *Å*. The rationale behind keeping constant distance between the virtual residue and the Cα is to test how bulkiness at positions close to the α-helix backbone affects the conformation of the α-helix, as in the case of ILE, VAL, and THR.

### Conceptual simulation 2: residues with far bulkiness

Here we repeat the same simulation described in Conceptual Simulation 1 but with an increased bond length of the virtual residue. We increased the distance of the virtual residue from the Cα and maintained it at *σ* − *1.96* *Å*. The rationale behind increasing the distance between the virtual residue and the Cα is to test how bulky residues like PHE (F), TYR (Y), and TRP (W) affect the conformation of the α-helix.

### Conceptual simulation 3: external electric field

The goal of this simulation is to demonstrate the dependency of the α-helical conformation on external electric field with cylindrical symmetry. To achieve the goal we applied an electric field with its zero set at the center of the α-helix and with an increasing linear potential extending outwards. The contribution of the electric potential energy was calculated as: *E*_*Electric*_ = *E*_*CO*_ + *E*_*NH*_, where *E*_*CO*_ = −*0.5·D*_*Helix*-*O*_ and *E*_*NH*_ = *0.33* · *D*_*Helix*-*H*_. *D*_*Helix*-*O*_ is the distance of the oxygen atom of the backbone carbonyl group from the α-helix center, and *D*_*Helix*-*H*_ is the distance of the estimated hydrogen atom of the backbone amide group from the α-helix center. The estimation of the hydrogen position assumed N-H distance of *0.98* *Å* (sampled from PDB), that the backbone atom positions of C, N, H, Cα are on the same plane, and equal bend angles C-N-H = Cα-N-H. The coefficients −*0.5* and *0.33*, are the partial electric charges of the backbone carbonyl oxygen and of the backbone amide hydrogen, respectively, according to OPLS.

### Conceptual simulation 4: external mechanical force

The goal of this simulation is to demonstrate the dependency of the α-helical conformation on homogeneous external stretching/squeezing forces that act on the α-helix residues from the α-helix center outwards for the stretching case, and towards the α-helix center in the case of squeezing. Other mechanical force that can act on the α-helix residues can be translated to an effective stretch/squeeze force. The contribution to the total energy of the applied mechanical force was calculated as: *dE*_*Stretch*_ = −*D*_*Helix*-*CA*_, where *D*_*Helix*-*CA*_is the distance of the backbone Cα from the α-helix center.

## Additional Information

**How to cite this article**: Haimov, B. and Srebnik, S. A Closer Look into the α-Helix Basin. *Sci. Rep.* **6**, 38341; doi: 10.1038/srep38341 (2016).

**Publisher's note:** Springer Nature remains neutral with regard to jurisdictional claims in published maps and institutional affiliations.

## Supplementary Material

Supplementary Information

Supplementary Dataset 1

Supplementary Dataset 2

Supplementary Movie 1

Supplementary Movie 2

## Figures and Tables

**Table 1:** Energy difference [EU] between homogeneous average conformations and measured conformation for transition AA_ROW_−>AA_COL_ for Level 1 filtering.

	A	R	N	D	C	Q	E	G	H	I	L	K	M	F	P	S	T	W	Y	V	Mean
A	**0**	0	1	0	0	0	0	0	1	0	0	0	0	2	14	0	1	0	2	0	1
R	1	**0**	1	3	0	0	0	1	1	1	0	0	1	4	11	0	1	1	0	1	1
N	4	2	**0**	1	3	2	1	6	8	4	2	2	3	16	19	3	1	4	12	4	5
D	0	0	1	**0**	0	0	0	1	0	1	0	0	0	6	12	1	1	0	3	1	1
C	5	2	4	3	**0**	2	4	3	3	1	2	4	2	2	20	1	0	1	2	1	3
Q	1	0	0	0	1	**0**	0	2	1	1	0	0	1	6	10	0	0	2	1	0	1
E	1	0	1	0	1	0	**0**	1	0	1	0	0	1	8	10	0	1	1	2	1	2
G	0	1	1	2	3	1	0	**0**	0	0	0	0	1	1	75	0	1	0	1	1	5
H	5	3	2	1	1	3	1	8	**0**	3	1	4	3	1	13	2	0	2	2	2	3
I	3	3	1	1	2	2	2	3	3	**0**	0	4	0	0	9	1	0	0	2	0	2
L	3	4	1	2	0	3	3	4	3	0	**0**	6	0	1	9	2	1	0	5	0	2
K	0	0	6	1	0	0	0	0	4	1	1	**0**	1	3	15	0	2	4	3	1	2
M	2	3	0	2	2	2	3	4	5	0	0	4	**0**	1	10	2	1	1	2	0	2
F	9	9	4	5	3	8	5	15	2	1	1	12	1	**0**	8	7	1	7	0	1	5
P	15	20	11	15	16	15	19	14	28	30	19	16	17	30	**0**	11	20	21	37	27	19
S	1	0	0	0	0	1	0	1	1	1	1	1	1	6	14	**0**	0	2	3	1	2
T	2	1	1	0	2	0	0	2	0	0	0	1	0	1	8	0	**0**	0	1	1	1
W	2	2	0	2	6	2	2	7	1	0	0	2	0	1	8	3	1	**0**	0	0	2
Y	7	7	4	4	4	4	2	13	0	1	1	8	2	0	10	5	0	7	**0**	1	4
V	4	3	1	1	1	2	2	3	2	0	0	4	0	1	10	1	0	0	2	**0**	2
Mean	3	3	2	2	2	2	2	4	3	2	2	3	2	5	14	2	2	3	4	2	3
