# Supplementary material for: A closer look into the α-helix basin
Source: Sci Rep. 2016 Dec 5;6:38341. doi: 10.1038/srep38341 (PMC5137006; doi:10.1038/srep38341)
Supplement: Supplementary Information [file srep38341-s1.pdf]

A closer look into the α-helix basin
Supplementary Information

Boris Haimov[1] & Simcha Srebnik[1,2,*]

[1] Russell Berrie Nanotechnology Institute, Technion - Israel Institute of Technology, Haifa, 32000, Israel

[2] Department of Chemical Engineering, Technion - Israel Institute of Technology, Haifa, 32000, Israel

* To whom correspondence should be addressed. Tel: +972-4-829-3584; Email: simchas@technion.ac.il

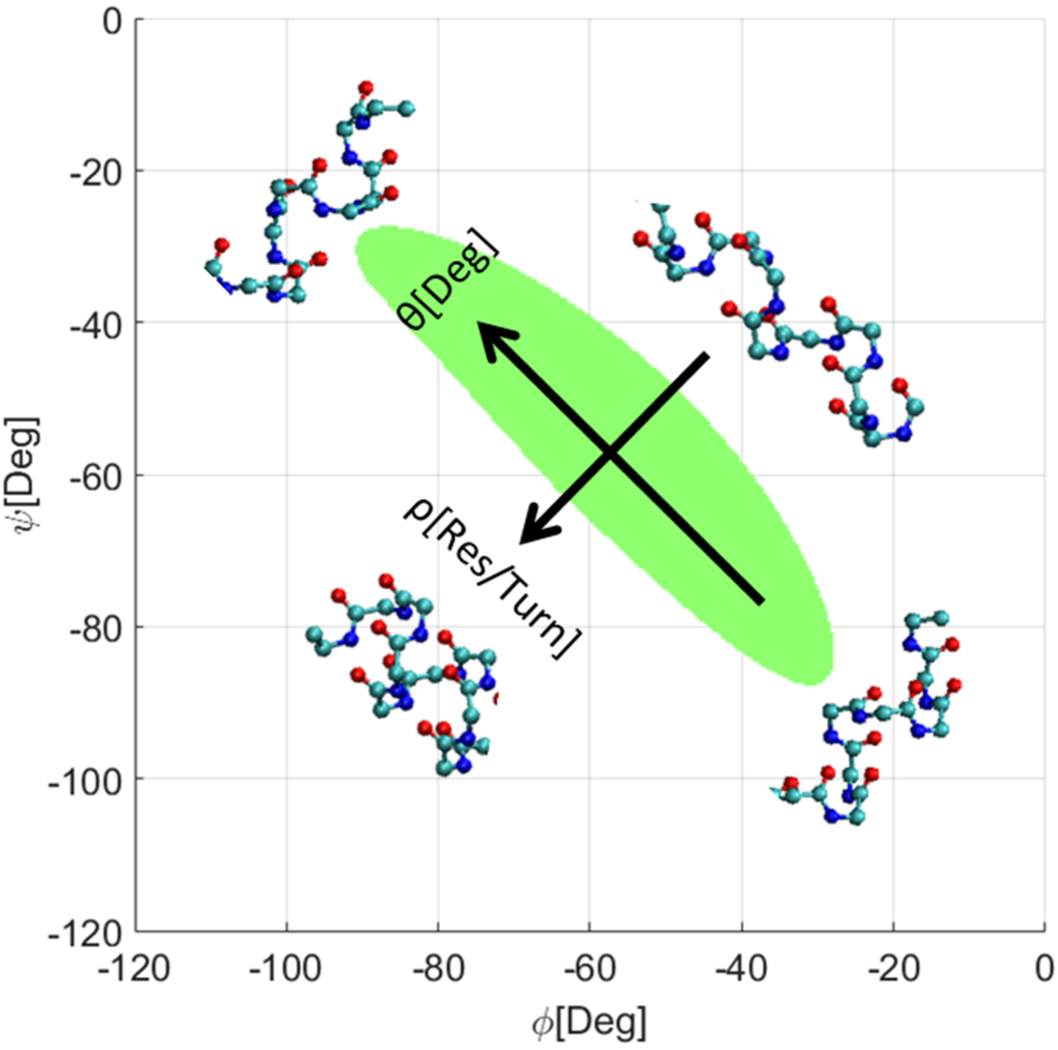

Supplementary Figure 1: α-helices showing representative regions of the Ramachandran map. Diagonals provide an alternative coordinates system (ρ,θ) for α-helices: ρ represents the amount of residues per turn, and θ represents the angle of backbone C=O groups with respect to the helix direction. Green region represents the α-helix basin determined by alignment of hydrogen bonds. PDB animations that demonstrate the change of ρ and θ in α-helices can be found in supplementary material SM1_rho.pdb and SM2_theta.pdb, respectively.

Supplementary Table 1: Energy difference [EU] between homogeneous average conformations and measured conformation for transition AA_{ROW}->AA_{COL} for Level 0 filtering

	A	R	N	D	C	Q	E	G	H	I	L	K	M	F	P	S	T	W	Y	V	Mean
A	0	0	1	1	0	0	0	4	0	0	1	0	0	2	18	0	1	1	1	0	1
R	1	0	2	3	0	0	0	3	1	2	1	0	1	6	18	0	2	0	1	1	2
N	9	3	0	2	3	2	2	3	6	5	4	2	6	20	31	3	1	8	12	6	6
D	2	0	2	0	1	0	1	8	1	1	1	1	1	10	35	1	0	3	2	0	3
C	6	5	2	4	0	3	4	7	6	2	2	7	1	4	45	3	0	0	3	2	5
Q	1	0	2	1	0	0	0	4	0	1	1	1	2	5	17	0	0	3	1	1	2
E	1	0	3	1	0	0	0	3	1	1	1	1	1	7	18	0	0	1	2	1	2
G	3	1	0	5	0	4	3	0	1	0	0	4	0	8	175	3	3	2	4	2	11
H	6	4	2	2	4	5	3	9	0	4	2	3	4	2	13	3	0	6	1	3	4
I	3	3	2	3	2	2	3	7	1	0	0	4	0	1	12	3	0	0	1	0	2
L	3	4	2	3	0	3	3	6	4	0	0	6	0	0	13	3	0	2	3	0	3
K	1	0	7	2	1	0	1	5	4	1	1	0	1	3	23	0	3	2	7	1	3
M	2	4	2	4	1	2	4	5	5	1	0	5	0	1	13	3	0	3	3	0	3
F	11	13	7	6	2	13	8	11	1	3	1	17	2	0	11	11	1	5	0	2	6
P	33	55	60	35	33	38	36	9	44	45	41	39	35	66	0	38	47	53	71	43	41
S	2	2	1	2	1	2	0	7	0	1	2	2	1	9	30	0	0	3	3	1	3
T	4	1	0	2	1	0	0	0	1	0	0	1	1	2	18	1	0	3	1	0	2
W	3	5	2	5	1	7	4	7	5	1	1	5	4	3	15	5	2	0	0	2	4
Y	7	9	3	4	2	4	3	8	1	1	1	9	2	0	13	7	1	4	0	1	4
V	4	3	1	3	1	2	2	7	1	0	0	5	0	2	13	2	0	1	1	0	2
Mean	5	6	5	4	3	4	4	6	4	3	3	6	3	8	26	4	3	5	6	3	6

Supplementary Table 2: Energy difference [EU] between homogeneous average conformations and measured conformation for transition AA_{ROW}->AA_{COL} for Level 2 filtering

	A	R	N	D	C	Q	E	G	H	I	L	K	M	F	P	S	T	W	Y	V	Mean
A	0	0	0	0	1	0	0	0	1	0	0	0	1	1	9	0	1	0	0	1	1
R	1	0	0	2	1	0	0	1	1	1	0	0	1	6	6	0	0	0	1	1	1
N	3	2	0	1	2	2	1	7	5	4	3	3	2	14	9	3	1	2	6	4	4
D	0	0	0	0	0	0	0	1	1	0	0	0	0	5	12	0	0	0	2	1	1
C	1	1	0	0	0	0	1	1	2	0	0	1	0	0	7	1	2	0	0	1	1
Q	1	0	0	0	1	0	0	3	1	1	0	0	1	4	6	0	0	1	1	1	1
E	1	0	0	0	2	0	0	1	2	1	0	0	1	6	5	1	1	0	2	1	1
G	1	2	3	3	2	2	1	0	1	0	1	0	1	0	66	0	1	0	0	2	4
H	2	1	1	0	2	1	0	5	0	2	1	3	4	3	7	1	2	0	4	1	2
I	2	1	0	1	0	1	1	5	2	0	0	2	0	1	5	1	0	0	1	0	1
L	2	2	1	2	0	1	1	3	1	0	0	2	0	2	5	2	1	0	2	0	1
K	0	0	1	0	1	0	0	1	1	1	1	0	0	4	9	1	1	2	0	1	1
M	2	2	1	2	0	2	1	5	3	1	0	1	0	2	6	1	2	1	1	0	2
F	5	5	2	3	0	6	3	10	0	0	0	5	0	0	6	3	2	18	0	0	3
P	16	18	12	11	15	14	18	19	32	24	16	16	19	23	0	11	22	24	29	26	18
S	1	0	0	1	0	0	0	1	2	0	0	0	0	4	9	0	0	2	1	0	1
T	1	0	0	0	0	0	0	3	0	0	0	0	0	3	4	0	0	0	0	1	1
W	2	1	0	2	1	2	2	5	1	0	0	3	0	0	3	3	2	0	0	0	1
Y	4	3	3	2	2	1	1	4	0	0	1	3	0	0	6	2	0	9	0	0	2
V	2	1	0	1	0	1	0	3	1	0	0	2	0	2	8	1	1	0	2	0	1
Mean	2	2	1	2	1	2	2	4	3	2	1	2	2	4	9	2	2	3	3	2	2

Supplementary Table 3: Energy difference [EU] between homogeneous average conformations and measured conformation for transition AA_{ROW}->AA_{COL} for Level 3 filtering

	A	R	N	D	C	Q	E	G	H	I	L	K	M	F	P	S	T	W	Y	V	Mean
A	0	0	0	0	0	0	0	0	1	0	0	0	0	1	8	0	1	0	0	1	1
R	1	0	0	2	0	0	0	1	1	0	0	0	1	4	4	0	0	0	1	0	1
N	2	1	0	1	3	1	1	6	4	3	2	2	1	13	7	2	1	0	5	3	3
D	0	0	0	0	1	0	0	1	1	1	0	0	1	4	8	0	0	0	2	1	1
C	2	1	0	1	0	1	2	1	2	0	0	0	0	1	4	0	2	0	0	0	1
Q	1	0	0	0	1	0	0	1	1	1	0	0	1	4	6	0	0	1	1	0	1
E	1	0	0	0	1	0	0	1	1	1	0	0	1	6	5	0	0	1	2	1	1
G	1	2	1	2	1	2	1	0	1	0	1	0	2	0	65	1	1	0	0	2	4
H	1	1	2	0	0	0	0	2	0	1	0	2	1	2	3	0	1	0	2	0	1
I	1	1	0	1	1	1	1	3	2	0	0	1	0	2	6	1	1	0	2	0	1
L	2	2	1	1	0	1	1	3	1	0	0	2	0	2	4	2	1	0	3	0	1
K	0	0	1	0	1	0	0	0	0	0	0	0	1	5	7	0	0	1	1	1	1
M	2	2	1	2	0	2	2	5	3	0	0	2	0	1	5	1	1	1	1	0	2
F	3	3	1	2	1	2	1	6	0	0	0	5	0	0	4	2	1	14	0	0	2
P	13	16	11	11	19	11	17	12	30	23	16	16	17	22	0	10	21	21	29	23	17
S	0	0	0	1	1	0	0	1	2	0	0	0	0	3	6	0	0	3	1	0	1
T	1	0	0	0	0	0	0	1	0	0	0	0	0	1	4	0	0	1	1	0	1
W	1	1	0	1	1	1	2	4	0	0	0	2	0	0	3	4	0	0	0	0	1
Y	3	2	2	2	1	0	1	6	0	0	0	3	0	0	3	1	1	8	0	0	2
V	1	1	0	1	0	0	0	2	1	0	0	1	0	2	7	1	1	0	2	0	1
Mean	2	2	1	1	2	1	2	3	3	2	1	2	1	4	8	1	2	3	3	2	2

Supplementary Movie 1: SM1_rho.mov file is a visualization of the dependency of the helical conformation on the amount of residues per turn (ρ axis). SM1_rho.pdb file is the PDB version of the animation.

Supplementary Movie 2: SM2_theta.mov file is a visualization of the dependency of the helical conformation on the CO angle (θ axis). SM2_theta.pdb file is the PDB version of the animation.
